# Mapping malaria vectors and insecticide resistance in a high-endemic district of Haryana, India: implications for vector control strategies

**DOI:** 10.1186/s12936-023-04797-8

**Published:** 2024-04-17

**Authors:** Gaurav Kumar, Sanjeev Gupta, Jaspreet Kaur, Shweta Pasi, Rajendra Baharia, Ajeet Kumar Mohanty, Pawan Goel, Amit Sharma, Manju Rahi

**Affiliations:** 1 ICMR-National Institute of Malaria Research, New Delhi, India; 2 ICMR-National Institute of Occupational Health, Ahmedabad, India; 3 Academy of Scientific and Innovative Research, Ghaziabad, India; 4 Shaheed Hasan Khan Mewati Government Medical College, Nuh, Haryana India; 5 International Centre of Genetic Engineering and Biotechnology (ICGEB), New Delhi, India; 6 Indian Council of Medical Research (ICMR), New Delhi, India; 7 ICMR-Vector Control Research Center, Puducherry, India

**Keywords:** Malaria, Elimination, Mosquito vectors, Insecticide resistance, Nuh Haryana

## Abstract

**Background:**

Achieving effective control and elimination of malaria in endemic regions necessitates a comprehensive understanding of local mosquito species responsible for malaria transmission and their susceptibility to insecticides.

**Methods:**

The study was conducted in the highly malaria prone Ujina Primary Health Center of Nuh (Mewat) district of Haryana state of India. Monthly entomological surveys were carried out for adult mosquito collections via indoor resting collections, light trap collections, and pyrethrum spray collections. Larvae were also collected from different breeding sites prevalent in the region. Insecticide resistance bioassay, vector incrimination, blood meal analysis was done with the collected vector mosquitoes.

**Results:**

A total of 34,974 adult *Anopheles* mosquitoes were caught during the survey period, out of which *Anopheles subpictus* was predominant (54.7%). Among vectors, *Anopheles stephensi* was predominant (15.5%) followed by *Anopheles culicifacies* (10.1%). The Human Blood Index (HBI) in the case of *An. culicifacies* and *An. stephensi* was 6.66 and 9.09, respectively. Vector incrimination results revealed *Plasmodium vivax* positivity rate of 1.6% for *An. culicifacies*. Both the vector species were found resistant to DDT, malathion and deltamethrin.

**Conclusion:**

The emergence of insecticide resistance in both vector species, compromises the effectiveness of commonly used public health insecticides. Consequently, the implementation of robust insecticide resistance management strategies becomes imperative. To effectively tackle the malaria transmission, a significant shift in vector control strategies is warranted, with careful consideration and adaptation to address specific challenges encountered in malaria elimination efforts.

## Background

Malaria remains one of the major causes of morbidity and mortality among vector-borne diseases. According to the World Malaria Report 2022, there were 247 million estimated cases and 619,000 deaths due to malaria, mainly in African nations (95% of total cases) [1]. The World Health Organization South-East Asia Region (WHO-SEARO) accounted for around 2% of the malaria burden worldwide. Malaria incidence in this region decreased significantly over the previous two decades, from 23 million in 2000 (18 cases per 1000 population) to around 5 million in 2021 (3 cases per 1000 population). India accounted for most of the malaria cases in WHO SEARO with 79% cases in the region [1]. Nonetheless, India has been able to sustain the decline in malaria burden over the years. The national malaria control programme reported a reduction in the number of cases from 1.1 million in 2015 to 0.16 million in 2021. However, at the same time, the data for the year 2021 revealed that around three-fourth of malaria cases recorded in India were confined to five states, Jharkhand, Maharashtra, Odisha, West Bengal, and Chhattisgarh [2].

The overall reduction in malaria burden in India has been attributed primarily to the scaling-up of control interventions, such as a prompt diagnosis by rapid diagnostics and microscopy, effective treatment, and importantly effective vector control via indoor residual spray (IRS) and long-lasting insecticidal nets (LLINs). According to the national guidelines, the distribution of LLINs targets 80% coverage with an average of one LLIN per 1.8 people in all sub-centers reporting an Annual Parasite Incidence of ≥ 1, whereas IRS is targeted to cover epidemic-prone areas and malaria-affected communities with low access to the health care system [3]. Vector control interventions have been at the forefront and contributed substantially to the reduction in malaria transmission.

In India, there are six primary malaria vectors, namely, *Anopheles culicifacies*, *Anopheles fluviatilis*, *Anopheles minimus*, *Anopheles stephensi*, *Anopheles baimaii* and *Anopheles sundaicus* and the secondary vectors are *Anopheles annularis*, *Anopheles philippinensis*, *Anopheles nivipes* and *Anopheles varuna* [4]. Studies have pointed out the emergence of secondary malaria vectors with a decline in *Anopheles minimus* and *Anopheles dirus* across Northeast of India [5, 6]. This underscores the critical need for precise data regarding species composition.

Vectors exhibit variable biological characteristics that influence their response to vector control measures. In the context of elimination, if the vector control interventions need to remain effective [7], entomological aspects, both physiological and behavioural, of the prevalent malaria vectors that influence malaria transmission are of paramount importance. Further, it would be appropriate to have recent data on bionomics and the efficacy of existing interventions on the vectors when assessing the effectiveness of current vector control tools and deciding which control strategy to be used in the field.

Haryana, a northern state of India, along with all its districts (the administrative unit of malaria control operations) was slotted in Category 1 with an Annual Parasite Incidence of less than 1 (API of 0.17 in 2014 of Haryana state) as per the National Framework for Malaria Elimination in India (2016–2030). The API of Haryana was 0.05 whereas Nuh district API was 0.68 in 2019. Nuh district reported 942 malaria cases in 2019 out of which 719 (API 9.8) were reported by Ujina Primary Health Centre area. Therefore, it was deemed important to understand the malaria vector distribution patterns and susceptibility to insecticides in highly malarious villages from the Ujina Primary Health Centre. In addition to rigorous vector surveillance, an epidemiological assessment of malaria situation through a mass survey was also conducted. This surveillance aimed to provide comprehensive insights into vector distribution, densities, vector incrimination, and their susceptibility to insecticides in the selected villages of Nuh district, Haryana. The findings of the study would be useful in planning suitable vector control strategies to control and eliminate malaria in the Haryana state of India.

## Methods

### Study sites

**Fig. 1:**
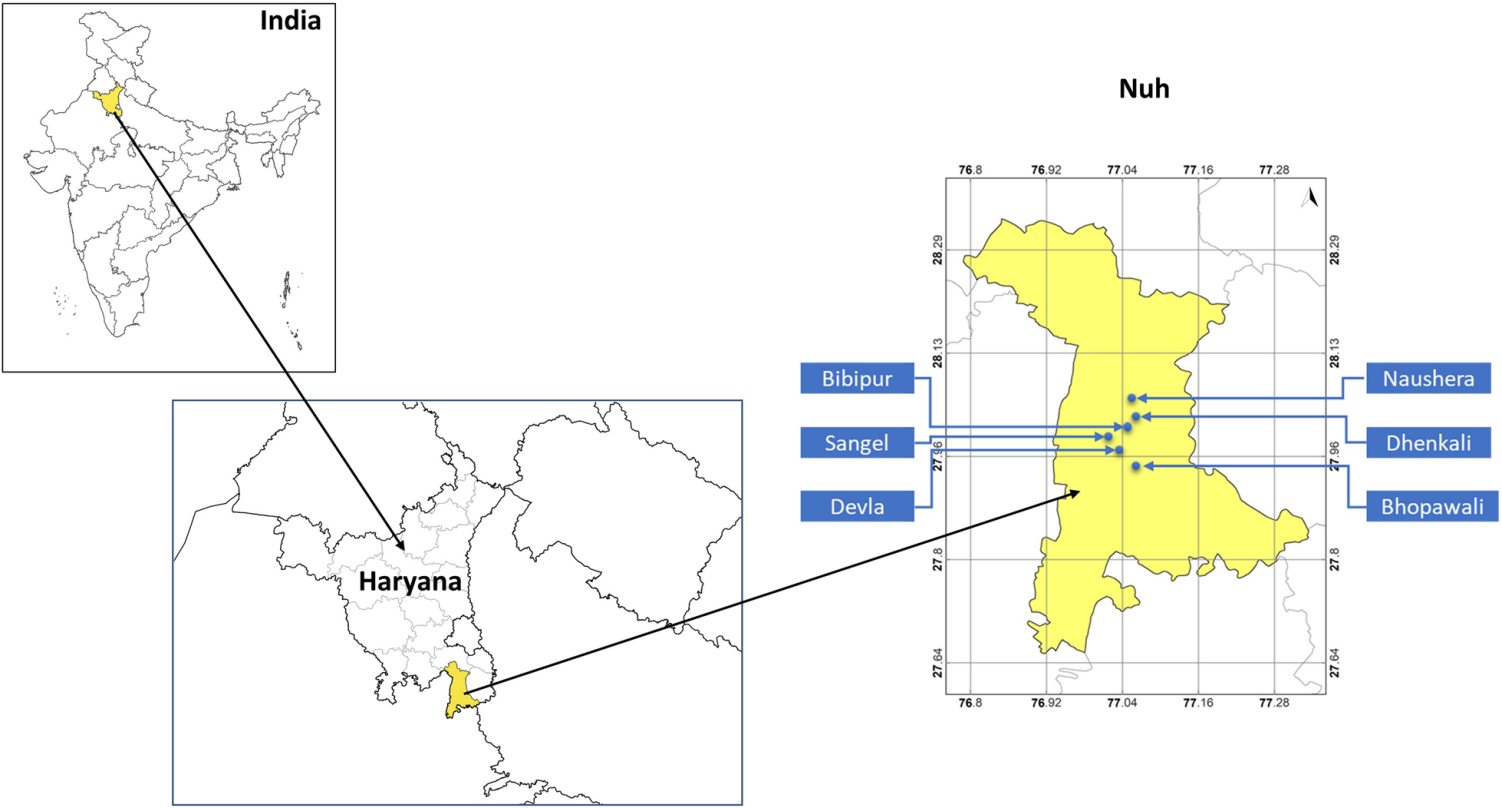
Study area with location of selected villages

The study was undertaken in district Nuh (Mewat) of Haryana state situated in the southern part of the state, bordering the neighbouring state of Rajasthan. The district Nuh is the most backward and malaria-endemic district of Haryana. In 2018, it was designated as the most underdeveloped district by NITI Aayog, the premier public policy think tank of the Indian government [8]. Nearly 95.3% population of Nuh district comes from rural areas and with a very low literacy rate (56%) compared to the national literacy rate (74%) as per census 2011 of India. The Nuh district comprises five blocks namely Nuh, Ferozpur Jhirka, Punhana, Taoru, and Nagina. Nuh block had the highest number of reported malaria cases in 2019 among the five blocks at the time of planning the study. The selected Ujina Primary Health Centre (PHC) had the highest API (9.8) among the ten PHCs of Nuh Block in 2019. Among the four sub-centers, namely Sangel, Bhaijera, Ujina, and Adbar under the Ujina PHC, Adbar reported the least malaria cases. We selected a total of six villages from 3 sub-centres i.e., Ujina, Sangel, and Bajhera based on the number of malaria cases reported in 2019 (Table 1). The geographical locations of all the selected villages is depicted in Fig. 1.

**Table 1:** Study area showing village-wise population and malaria cases

Villages (Sub-centre)	Population	Malaria cases (2019)
Bibipur (Ujina)	3734	92
Bhopawali (Bajhera)	1643	85
Naushera (Sangel)	4438	88
Sangail (Sangel)	4119	68
Devla (Bhajhera)	6658	99
Dhenkali (Bhajhera)	2494	45

Entomological surveys were carried out to understand vector distribution, densities, vector incrimination, and their susceptibility to insecticides. Monthly collections were made from July 2021 to May 2022.

### Collection of larvae and adult mosquitoes

The different methods used for the collection of adult anopheline mosquitoes from the selected villages were as follows:

#### Indoor resting collections

*Anophelines* resting indoors were caught by hand using a mouth aspirator and a flashlight. In each village, four human dwellings (HD) and four cattle sheds (CS) were searched for mosquitoes in the morning from 06:00 to 08:00 h, spending 15 min in each structure [9]. The standard morpho-taxonomic keys were used to identify mosquito species collected from the field [10]. From the number of female mosquitoes, the per man-hour density (MHD) of each vector species was computed.

#### Light trap collections (CDC light trap)

Adult vector density was also monitored using light traps which were placed inside human dwellings near eaves, sleeping hosts, and door for mosquito collection from 18:00 to 06:00 h. The mosquitoes were transported in an insulated box with wet towel at the bottom and outside. Further the vehicle used for transportation was air conditioned to make the temperature optimal for mosquito survival. From the light trap collections, vector density i.e. number of females collected per trap night indoors or outdoors was calculated.

#### Pyrethrum spray collection (PSC)

Pyrethrum spray collection (PSC) was another method used to collect adult mosquitos that were resting indoors. Most, if not all, of the *Anopheles* mosquitoes resting indoors, were collected using this procedure in the morning hours (08:00–10:00 h). The entire floor of the room (human dwelling) was covered with a white cotton sheet, and by spraying the complete room with 0.1–0.2% pyrethrum spray with a flit sprayer, all mosquitoes resting inside the room were knocked down onto the sheet. The mosquitoes collected were kept in petri dishes lined with wet cotton or filter paper and transported to the laboratory. This method gave the total number of mosquitoes/species resting per structure.

#### Breeding site surveys

Breeding site surveys were carried out in all villages covering all water collections within a radius of 500 m of the villages. The larvae collection was carried out around the study villages following the standard method. Various breeding sites such as ponds, pools, paddy fields, borrow pits, cement tanks, freshwater drains, rainwater collection, etc. were searched for the presence of *Anopheles* larvae. Collected larvae were reared and allowed to emerge into adults which were identified as per standard identification key [10].

### Laboratory processing

#### Mosquito species identification

Mosquitoes collected by different methods were morphologically identified to different *Anopheles* species using the taxonomic keys of Nagpal and Sharma [10].

#### Abdominal condition and blood meal preferences

The physical condition of vector mosquitoes’ abdomen was observed under a microscope and mosquitoes were categorized as unfed, full-fed, semi gravid, and gravid according to their abdominal status [7]. Blood from the stomach of the fully-fed mosquitoes obtained from the field was collected on Whatman No. 1 filter paper to identify the source of blood meal against human and bovine anti-sera human blood index (HBI) of *An. culicifacies* and *An. stephensi* was determined using the polymerase chain reaction (PCR). Each blood spot was processed for the isolation of genomic DNA using a Qiagen kit (Qiagen, Germany). The source of blood meal was detected using the multiplex PCR as previously described [11].

#### Vector incrimination

Mosquitoes obtained from different collection methods were examined for vector infection with human malaria parasites. The head and thoraces of the mosquitoes were dissected and stored in isopropanol at − 20 °C until use. Vector incrimination was done by the ELISA-based method to detect species-specific circumsporozoite antigen (CSP) of *Plasmodium falciparum*, *P. vivax* 210, and *P. vivax* 247 using the protocol described by Wirtz et al. [12, 13].

### Detection of *Plasmodium* infection in the mosquito abdomen using nested PCR

In the first step, the presence of *Plasmodium* infection in the mosquito abdomen was detected using the genus-specific universal primers. Further, the amplified PCR product was utilized to detect the species of Plasmodium using nested PCR [14].

#### Insecticide susceptibility tests

Vector susceptibility to the insecticides used in the national control programme was tested once during the peak abundance of vector species, following the WHO guidelines [15]. Field collected, preferably from unsprayed villages/houses, mixed-age vector mosquitoes were exposed to the WHO papers impregnated with insecticides at the diagnostic concentration (DDT 4%, malathion 5%, similarly WHO-recommended discriminating concentration of different synthetic pyrethroids) using a WHO test kit. For a test, 100 mosquitoes, in four/five replicates (20–25 mosquitoes per replicate) for treatment and 50, in two replicates (25 mosquitoes per replicate), for control were exposed. Tests were carried out in a room with no insecticide contamination and maintained at 27 ± 2ºC temperature and 80 ± 10% relative humidity both during exposure and during 24 h holding period. From the total number of alive and dead mosquitoes in the replicates, the percent mortality was calculated post-24 h of holding. If control mortality was between 5% and 20%, Abbott’s formula was used to correct the treatment mortality. According to WHO criteria, mortality of ≥ 98% was considered to be ‘susceptible’; <90%—‘resistant’; and between 91 and 97%—‘possible resistance’.

### Data analysis

All data generated during the study were entered into MS Excel, and the following parameters were analysed.

- Man-hour density is the number of mosquitoes collected by one person for one hour and is calculated taking into account the total number of mosquitoes (n) collected, time spent in minutes (t), and the number of persons involved in the collection (p). MHD = n × 60/t × p.
- The proportion of fed *Anopheles* mosquitoes found to contain human blood was used to calculate the human blood index (HBI).
- The proportion of female *Anopheles* mosquitoes carrying sporozoite in their salivary glands was used to calculate the sporozoite rate.
- Map was prepared using QGIS, an open-source geographic information system (QGIS Development Team, 2022) software.

### Statistical analysis

The data was analysed using descriptive (mean and standard deviation) and inferential statistics (comparison of means), and chi-squared tests were used to assess the association between dependent and independent variables. During analysis P-value, less than or equal to 0.05 was considered statistically significant. The analysis was performed using the SPSS® Statistics program (IBM Corporation, Armonk, NY, USA).

#### Ethical clearance

The study is a collaborative effort between the Indian Council of Medical Research-National Institute of Malaria Research (ICMR-NIMR, New Delhi), the International Centre for Genetic Engineering and Biotechnology (ICGEB, New Delhi), and Shaheed Hasan Khan Mewati (SKHM) Government Medical College, Nuh (Haryana). An approval from the Institutional Ethical Committee of ICMR-NIMR (NIMR/EC/2021/08 dated 21.12.2021) was obtained.

## Results

### Summary of adult mosquito collection

A total of 68,986 adult mosquitoes were caught during the survey period from July 2021 to May 2022 out of which 34,974 (50.7%) were *Anopheles*, 541 (0.8%) were *Aedes*, and the remaining 33,471 (48.5%) were *Culex*. The species composition of six *Anopheles* captured was *An. culicifacies* (10.1%), *An. stephensi* (15.5%), *An. subpictus* (54.7%), *Anopheles vagus* (7.8%), *Anopheles pulcherrimus* (4.2%), *Anopheles nigerrimus* (1.8%), and *An. annularis* (6.0%). A total of 18,923 (54.1%) *Anopheles* were collected using the resting collection method (human dwellings/cattle sheds), whereas 6,836 *Anopheles* (19.5%) were collected using the total catch method, and the remaining 9215 *Anopheles* (26.3%) were collected using light traps (indoor/outdoor). Among six villages in the study area, collected *Anopheles* were 5368 (15.3%), 6561 (18.8%), 4185 (12.0%), 8260 (23.6%), 5042 (14.4%), and 5558 (15.9%) from Bhopawali, Bibipur, Devla, Dhenkali, Naushera, and Sangel, respectively. The two known vector species of the region *An. culicifacies* (ranging between 3.9% and 16.2% of total *Anopheles* collected) and *An. stephensi* (ranging between 6.4% and 36.3% of total *Anopheles* collected) were captured from all villages (Table 2).

**Table 2:** Summary of adult mosquitoes collected from the study site

	Bhopawali		Bibipur		Devla		Dhenkali		Naushera		Sangel		Total	
	n	%	N	%	n	%	n	%	n	%	n	%	n	%
*An. culicifacies*	571	10.6	665	10.1	164	3.9	1337	16.2	580	11.5	208	3.7	3525	10.1
*An. stephensi*	613	11.4	2382	36.3	570	13.6	530	6.4	380	7.5	936	16.8	5411	15.5
*An. subpictus*	3661	68.2	2645	40.3	2988	71.4	3462	41.9	3003	59.6	3388	61.0	19,147	54.7
*An. vagus*	69	1.3	36	0.5	62	1.5	2346	28.4	92	1.8	114	2.1	2719	7.8
*An. pulcherrimus*	175	3.3	263	4.0	172	4.1	174	2.1	277	5.5	393	7.1	1454	4.2
*An. nigerrimus*	83	1.5	139	2.1	63	1.5	90	1.1	174	3.5	82	1.5	631	1.8
*An. annularis*	196	3.7	431	6.6	166	4.0	321	3.9	536	10.6	437	7.9	2087	6.0
Total *Anopheles*	5368	51.9	6561	43.5	4185	57.3	8260	54.6	5042	51.4	5558	49.1	34,974	50.7
Total *Aedes* spp.	14	0.1	29	0.2	310	4.2	34	0.2	120	1.2	34	0.3	541	0.8
Total *Culex* spp.	4970	48.0	8494	56.3	2803	38.4	6837	45.2	4644	47.4	5723	50.6	33,471	48.5

### Relative abundance of species according to the collection method

**Fig. 2:**
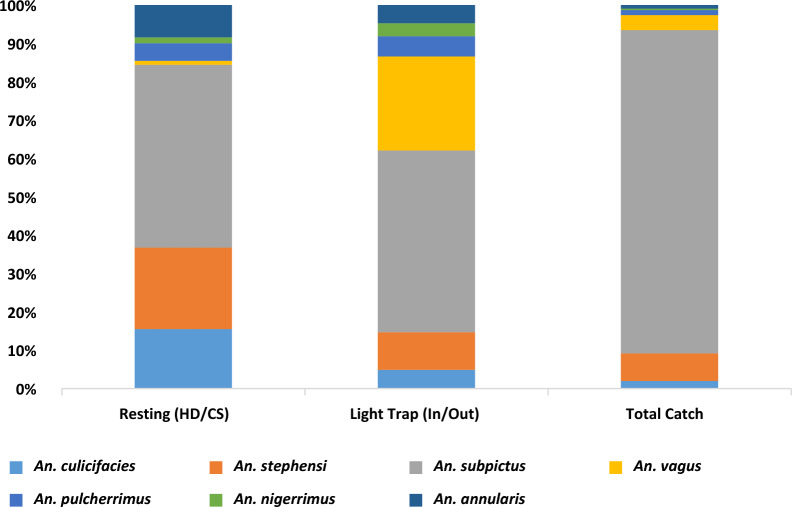
Relative abundance (%) of mosquito species according to the collection method (*HD* Human Dwelling, *CS* Cattle shed)

Figure 2 depicts the relative abundance of mosquito species varied according to the collection method. Adult mosquito collections through resting collection methods (human dwellings/cattle sheds) were in man-hour density (MHD), whereas collections via light traps (indoor/outdoor) were in mosquitoes collected per trap per night, and total catch collections were in mosquitoes collected per structure. In all collection methods, *An. subpictus* had the highest proportion, about 47% each by resting and light trap method, and 84% by total catch method. *Anopheles culicifacies* (15.5%) and *An. stephensi* (21%) were captured higher in the resting collection method than light trap and total catch collection methods (Fig. 2). Light trap collection method yielded mainly *An. subpictus* (47.4%), *An. vagus* (24.5%), *An. stephensi* (9.8%), *An. pulcherrimus* (5.3%), *An. culicifacies* (4.9%) and *An. nigerrimus* (3.4%). Total catch collection yielded mainly *An. subpictus* (84.4%) and *An. stephensi* (7.2%), the rest of the *Anopheles* species were in a small proportion.

The mean MHD of *Anopheles* collected by resting method in human dwellings/cattle sheds was 71.7 ± 61.9 (Mean ± SD), via light traps indoors/outdoors mean density was 69.8 ± 186.1 *Anopheles* per trap per night, and via total catch, the mean density was 103.6 ± 260.2 *Anopheles* per structure. The data exhibited a wide range of values, ranging from zero to extremely high numbers, leading to significantly higher standard deviations across all methods. These larger standard deviations indicate a deviation from normality in the data. Therefore, non-parametric tests were carried out. The MHD of mosquitoes resting in cattle sheds (97.8 ± 70.2) had found significantly higher than in human dwellings (45.6 ± 37.5) (Mann-Whitney U = 1116.5, P < 0.001). But there were no statistical differences on whether light traps were placed indoors (68.9 ± 219.1 mosquitoes/trap-night) or outdoors (70.7 ± 147.6 mosquitoes/trap-night) (Mann–Whitney U = 2066, P = 0.61).

### Relative abundance of species according to location

**Fig. 3:**
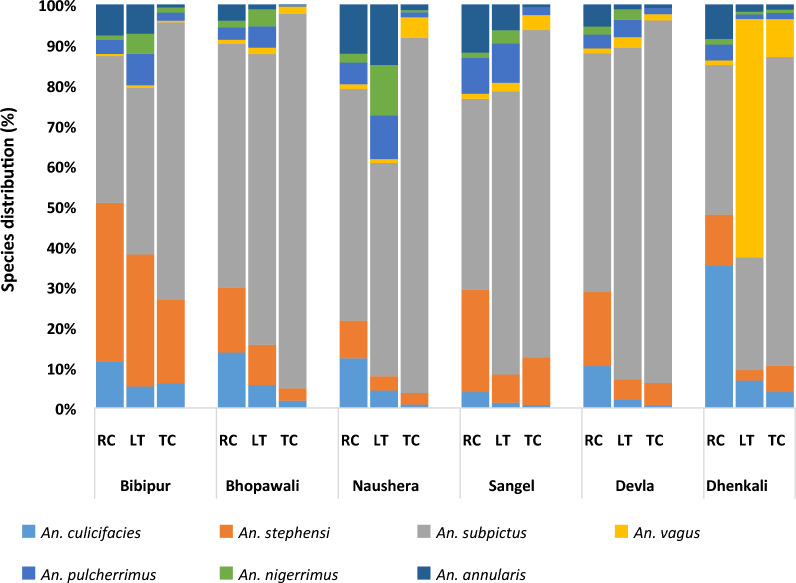
Relative abundance of the adult mosquito species caught by all three collection methods geographically (*RC* resting collection, *LT* light trap, *TC* total catch)

The relative abundance of the different mosquito species, measured as a percentage of total numbers caught by all three collection methods varied geographically (Fig. 3). A Kruskal–Wallis H test showed that there was a statistically significant difference in adult *Anopheles* collected from different villages, χ^2^(5) = 20.941, p = 0.001, with a mean rank of 199.54 for village Bibipur, 192.19 for Dhenkali, 168.14 for Sangel, 155.41 for Naushera, 144.53 for Bhopawali, and 132.2 for Devla village. Among vector species, village Dhenkali had a predominance of *An. culicifacies* followed by *An. stephensi*, whereas all other villages had a predominance of *An. stephensi* followed by *An. culicifacies*. In Bibipur and Dhenkali villages, the proportion of vector collection by resting collection method was ~ 50% of the total *Anopheles* collected.

In the case of non-vector species, *An. subpictus* was the predominant species found in all villages. However, in Dhenkali village, *An. vagus* was also captured in relatively high numbers, while in Naushera village, *An. nigerrimus* was captured in high proportions through light trap collection method.

### Abdominal condition of vector species

**Fig. 4:**
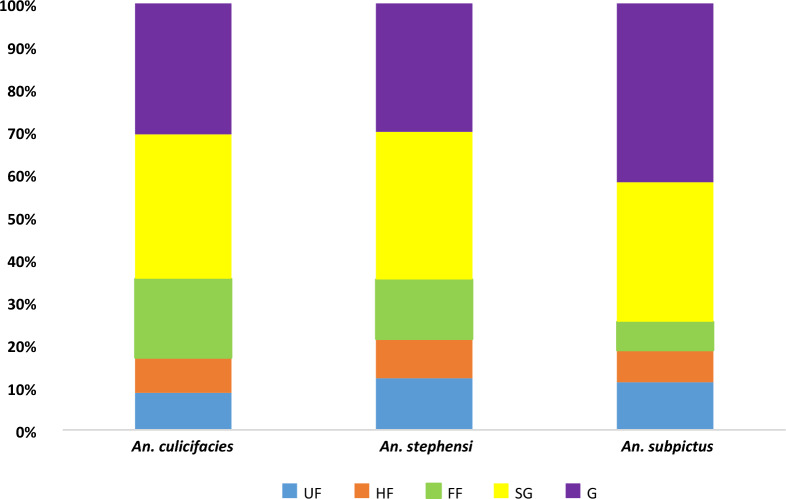
Abdominal status of mosquitoes collected at Nuh (*UF* unfed, *HF* half fed, *FF* full fed, *SG* semi gravid, *G* gravid)

In all the study villages, in indoor human dwelling collections (hand catch and pyrethrum spray collections), the proportion of semigravids (SG) plus gravids (G) of *An. culicifacies* was 64.5%. Similarly, the proportion of semigravids (SG) plus gravids (G) of *An. stephensi* was 64.7% and for *An. subpictus*, it was 74.6%. These data suggest a predominantly endophilic behaviour of *An. culicifacies, An. stephensi* and *An. subpictus* in six of the study villages (Fig. 4).

### Detection of blood meal source in vector species

The source of blood meal in *An. culicifacies* and *An. stephensi* was detected using multiplex PCR. The human blood index (HBI) in the case of *An. culicifacies* and *An. stephensi* was 6.66 and 9.09 respectively as shown in Table 3. The proportion of *An. stephensi* mosquitoes found positive for feeding on cow blood was 21.81%. Whereas, none of the *An. culicifacies* found positive to feed on cow blood.

**Table 3:** Detection of blood meal source in the abdomen of *Anopheles stephensi* and *Anopheles culicifacies* using multiplex PCR

Mosquito species	Number of samples tested	Number of samples positive for human blood	Number of samples positive for cow blood	% Human Blood Index (HBI)
*An. stephensi*	55	5	12	9.09
*An. culicifacies*	60	4	0	6.66

### Larval survey

**Fig. 5:**
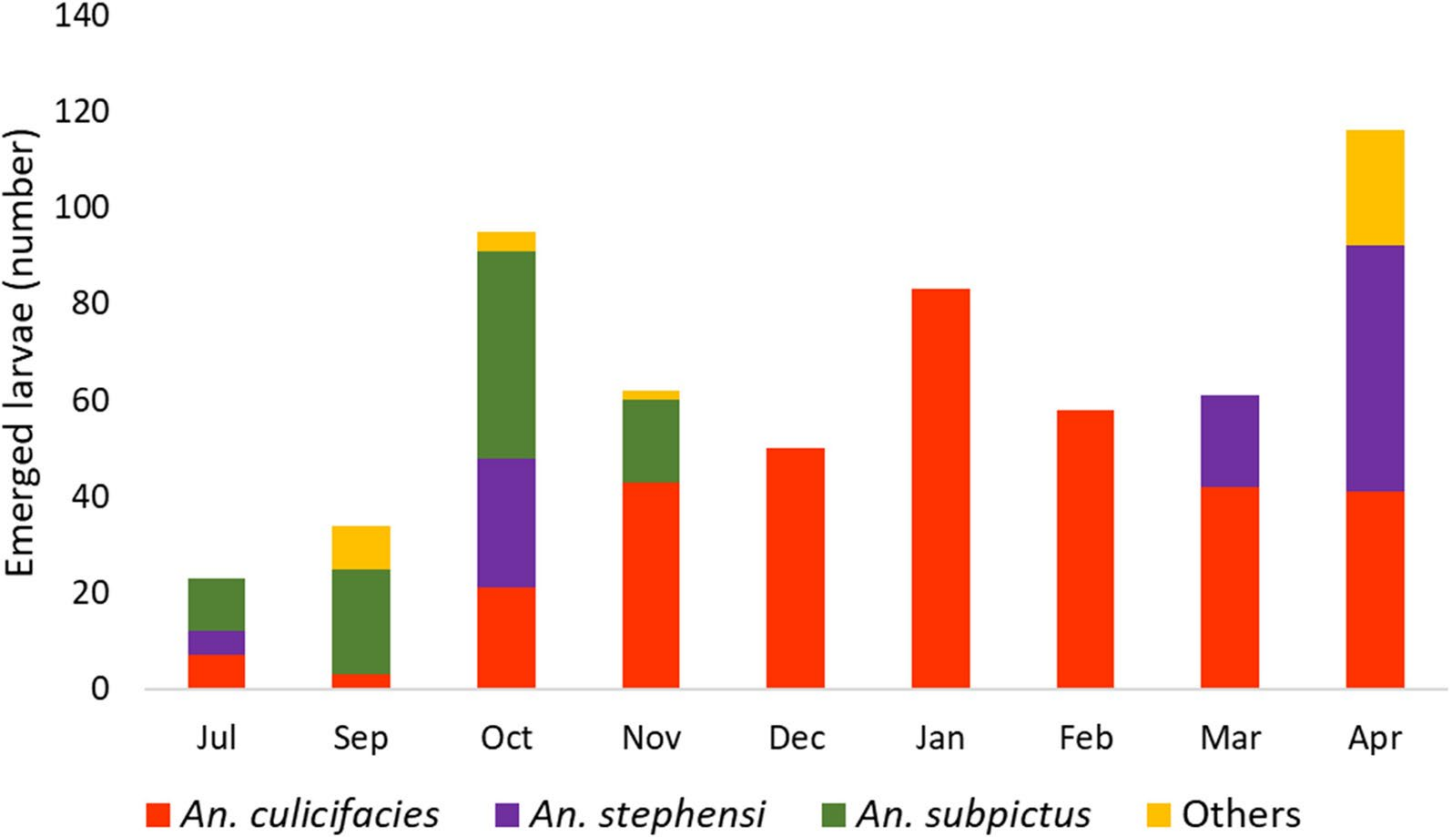
Monthly distribution of emergence of mosquito larvae at Nuh

During the study period, a total of 5 anopheline species were identified from different breeding habitats (ponds, pools, rice fields, ditches, pits, cemented tanks, underground tanks, desert coolers, and wells). *An. culicifacies* emerged in more numbers (75%) in comparison to other species *An. stephensi* (14.5%), *An. subpictus* (5.5%) and other mosquitoes (4.7%). During the monsoon season, the emergence of *An. subpictus* was more while *An. culicifacies* emergence was more during the post-monsoon and winter seasons (Fig. 5).

### Vector incrimination

The ELISA assay result for vector incrimination revealed zero positivity for sporozoite in the 692 *An. culicifacies* and 1673 *An. stephensi* tested mosquitoes. The presence of *Plasmodium* infected blood meal was detected in the abdomen of fully-fed *An. stephensi* and *An. culicifacies* using nested PCR. One out of 60 *An. culicifacies* samples processed were found positive to feed on *P. vivax* patient blood.

### Vector susceptibility to insecticides

*Anopheles culicifacies* was resistant to DDT (30.7–58% mortality) and malathion (44–72.5% mortality) in all the study villages (Table 4). It was resistant to deltamethrin in four villages Bibipur (79.2% mortality), Bhopawali (64% mortality), Naushera (78% mortality), and Devla (78.9% mortality) but showed possible resistance in Dhenkali (93% mortality). In all the study villages, *An. stephensi* was found resistant to DDT (21.5–57.4% mortality), malathion (44–52%), and deltamethrin (73–86.2% mortality) (Table 5).

**Table 4:** Percent mortality of *Anopheles culicifacies* against different insecticides in selected villages of Nuh

District	DDT (4%)			Malathion (5%)			Deltamethrin (0.05%)		
	Mosquito tested (n)	% mortality	Susceptibility status^a^	Mosquito tested (n)	% mortality	Susceptibility status	Mosquito tested (n)	% mortality	Susceptibility status
Bibipur	101	39.6	R	103	62.1	R	101	79.2	R
Bhopawali	100	53	R	101	62.3	R	100	64	R
Dhenkali	100	58	R	102	72.5	R	100	93	PR
Devla	75	37.3	R	75	44	R	76	78.9	R
Naushera	52	30.7	R	50	56	R	50	78	R

^a^≥98–100% mortality: susceptible (S); 90–97% mortality: possible resistance (PR); <90% mortality: resistant (R)

**Table 5:** Percent mortality of *Anopheles stephensi* against different insecticides in selected villages of Nuh

District	DDT (4%)			Malathion (5%)			Deltamethrin (0.05%)		
	Mosquito tested (n)	% mortality	Susceptibility status^a^	Mosquito tested (n)	% mortality	Susceptibility status	Mosquito tested (n)	% mortality	Susceptibility status
Bibipur	102	53.1	R	101	44	R	100	82.6	R
Bhopawali	102	29.6	R	100	44.5	R	101	73	R
Dhenkali	101	57.4	R	100	52	R	101	84.1	R
Devla	50	32	R	51	47	R	51	78.4	R
Naushera	51	21.5	R	51	45.1	R	51	86.2	R

^a^≥98–100% mortality: susceptible (S); 90–97% mortality: possible resistance (PR); <90% mortality: resistant (R)

## Discussion

Understanding the distribution of malaria vectors and their susceptibility to insecticides is essential for effective vector control measures. It enables targeted interventions, facilitates insecticide resistance management, guides the selection of appropriate control strategies, and supports adaptive management of vector control programmes. Ujina PHC of District Nuh in Haryana state is a malaria epidemic-prone area in India. In this surveillance study, crucial findings are presented regarding vector distribution, densities, vector incrimination, and their susceptibility to insecticides in six villages of Ujina PHC. The results of the study revealed that two malaria vector species *An. culicifacies* and *An. stephensi* are prevalent in the region. The vector species *An. culicifacies* (15.5%) and *An. stephensi* (21.0%) were captured higher in the resting collection than by the light trap and total catch collections. In a study conducted in Odisha, the density of *An. culicifacies* was lesser by light trap collections than the indoor resting collection density [16]. The overall prevalence of *An. stephensi* was more (15.5%) as compared to *An. culicifacies* (10.1%). In India, *An. stephensi* exists in three distinct biological forms i.e. mysorensis, type form, and intermediate form. Within Nuh, mysorensis and intermediate forms of *An. stephensi* were present and the type form was not found in the current study of mosquito collections. The ELISA assay result for vector incrimination revealed zero positivity for sporozoite in the 692 *An. culicifacies* and 1673 *An. stephensi* tested mosquitoes. However, one of the fully blood-fed *An. culicifacies* were found positive for *P. vivax* infected blood meal through PCR, which indicates that a very low level of malaria transmission is happening in the study sites. The HBI of *An. stephensi* was found comparatively higher than the *An. culicifacies*. However, the higher proportion of *An. stephensi* was found to prefer to feed on cow blood as compared to human blood. HBI of *An. culicifacies* was observed to be higher than that reported in other states [16, 17]. HBI variation may be due to different factors that may impact host availability in different settings.

In India, *An. stephensi* mysorensis and *An. stephensi* intermediate has been classified as poor vectors of malaria [4, 18] and, the vector incrimination findings are a confirmation of these facts. The *An. subpictus* mosquito constituted more than half of the collected mosquitoes in the study area. Therefore, vector incrimination studies on *An. subpictus* are required which could not be undertaken in the current study. Recently, role of secondary or other non vector species has been found in malaria transmission. Studies have pointed out towards role of *An. phillipinensis*, *An. nivipes*, *An. annularis*, *An. culicifacies* and *An. vagus* in malaria transmission across Northeasten states of India [5, 6]. Similarly, *An. subpictus* has been reported *Plasmodium* positive in natural collections from Ghaziabad (Uttar Pradesh) and Goa [14, 19]. Previously it has been found *Plasmodium* positive from Odisha, West Bengal and Tamil Nadu states of India [20]. In all the study villages, the proportion of semi-gravids (SG) plus gravids (G) of *An. culicifacies* and *An. stephensi* was > 60% indicating predominantly endophilic behaviour of malaria vectors in Nuh. Generally, *An. culicifacies* is an endophilic species in India but some recent reports have indicated towards change in the resting behaviour of this vector mosquito [21, 22].

Both the vector species *An. culicifacies* and *An. stephensi* were resistant to DDT, malathion, and deltamethrin in the Nuh district. In a previous report from the same district, *An. culicifacies* was found under the verification required category while *An. stephensi* was resistant to deltamethrin [23]. The findings of the current study showed that the frequency of resistance in both the malaria vectors is increasing in the study area. It may be due to the use of deltamethrin in IRS programme as well as the distribution of pyrethroids insecticide-based LLINs in the region. It suggests a change in the insecticide class usage both in IRS and LLINs. As per the recommended strategy, the same class of insecticide should not be used in both IRS and LLIN, rather a different class of insecticide should be used to avoid cross-resistance. To overcome the pressing issue of resistance development against the available insecticide in use, a new generation of LLIN incorporating either piperonyl butoxide (PBO) or novel insecticides might be beneficial [24, 25]. Another strategy could be the possibility of attractive targeted sugar baits application which has shown its efficacy under laboratory settings against both *An. culicifacies* and *An. stephensi* [26–28].

Comprehensive understanding of local mosquito species responsible for transmitting malaria and their susceptibility to insecticides is crucial for effectively controlling and eliminating malaria from malaria endemic region such as Nuh district of Haryana. Regular entomological collection of data is essential in elimination programmes to guide vector control strategies and evaluate their impact on malaria transmission. The predominant vector species in the region is *An. culicifacies*, highlighting the need for vector control strategies specifically targeting this species. Although *An. stephensi* was found in the area, it did not appear to play a significant role as a vector. However, additional research should be conducted to fully understand the potential involvement of *An. stephensi* in malaria transmission. Furthermore, the current study uncovered the development of insecticide resistance in both vector species, affecting all commonly used public health insecticides. Consequently, it is essential to implement effective insecticide resistance management strategies. These strategies should aim to address the issue of resistance and ensure that the chosen insecticides remain effective in controlling the vector populations. The findings underscore the significance of entomology in the context of malaria elimination goal of India. To effectively address residual malaria transmission, a significant shift in vector control strategies is necessary, and specific challenges related to malaria elimination should be carefully addressed and adjusted accordingly.

## Conclusion

Malaria control relies heavily on vector control. Therefore, it is extremely crucial to routinely follow the dynamics of vector populations and their behaviour towards applied insecticides in the form of IRS and LLINs. The present study provides important insights into the vector distribution and the insecticide susceptibility status of the major malaria vectors in Nuh, an epidemic prone district in the state of Haryana, India where IRS is routinely practiced and LLIN distribution is also being implemented for vector control. Despite vector control measures in place in the study area, it was striking to note a high density of *Anopheles* mosquitoes, primarily *An. subpictus*, and a significantly high human blood index among the major malaria vectors. This information underscores the urgency of closely monitoring vector control operations. Additionally, the presence of insecticide resistance against pyrethroids among the local vector populations raises alarm to scrutinize the vector control strategies and their implementation in the area. As India rapidly paces towards its deadline for malaria elimination, such studies are pressing-priorities for fine-tuning vector control strategies especially in endemic-regions for effective vector control and achievement of the goal of elimination in a time-bound manner.

## Data Availability

The study data are available by request to the corresponding author.
